# Right External Carotid Artery Originated Right Superior Thyroid Artery in Cadavers of a Medical College in Nepal: A Descriptive Cross-sectional Study

**DOI:** 10.31729/jnma.6419

**Published:** 2021-09-30

**Authors:** Nitasha Sharma, Ruku Pandit, Bhima Neupane, Ram Prakash Sah, Laxmi Bhattarai, Pranav Kumar Yadav

**Affiliations:** 1 Department of Anatomy, Universal College of Medical Science and Teaching Hospital, Bhairahawa, Nepal; 2 Department of Anatomy, College of Medical Science and Teaching Hospital, Bharatpur, Nepal; 3 Department of Anatomy, Manipal Medical Science and Teaching Hospital, Pokhara, Nepal

**Keywords:** *cadavers*, *external carotid artery*, *superior thyroid artery*

## Abstract

**Introduction:**

External carotid artery originated superior thyroid artery are frequently documented in classical anatomical, surgical and radiological textbooks. Knowledge of anatomical variations, proper identification of superior thyroid arteries is very important to avoid major complications during and after neck surgeries. The aim of the study is to find out the prevalence of right superior thyroid artery originating from right external carotid artery in cadavers of a Medical College in Nepal.

**Methods:**

A descriptive cross-sectional study was carried out at the department of anatomy in Universal College of Medical Sciences, Bhairahawa, Nepal from October 2020 to January 2021 after ethical clearance from the same institution (IRC UCMS, Ref: UCMS/IRC/078/20). Variations in origin of superior thyroid arteries were observed, recorded and photographed. Convenient sampling method was used. Data was analyzed using Microsoft Excel 2016. Point estimate at 90% Confidence Interval was calculated along with frequency and percentage.

**Results:**

Out of 30 right superior thyroid arteries of 30 cadavers, 27 (90%) at 90% Confidence Interval (80.22-96.44) originates from right external carotid artery.

**Conclusions:**

In our study we observed that almost nine tenths of right superior thyroid arteries originated from the right external carotid artery which was relatively high in comparison to other studies. Thus, Extensive knowledge of variations in origin of superior thyroid artery is important for surgeons prior to various interventional surgeries.

## INTRODUCTION

Superior thyroid artery (STA) is one of the large vasculature of head and neck region supplying larynx and thyroid gland.^1^ It is originated from external carotid artery (ECA) but occasionally it also originates from common carotid artery and carotid bifurcation.^2^ Variations in the right external carotid artery originating from superior thyroid arteries are frequently documented in classical anatomical, surgical and radiological textbooks.^4-5^ Ample knowledge about variations in arterial supply of superior thyroid artery is required prior to formulating planned neck surgeries and in alerting the surgeons to avoid inadvertent injuries to the vital anatomical structures in this area.^6^

Additionally, a detailed knowledge of these explicit arterial variations is extremely helpful while carrying out procedures like carotid angiographies, neck dissections and thyroid resections.^3^ Knowledge of such variations of origin superior thyroid artery has immense importance in academic and clinical arena.^7^

This study aims to find out the prevalence of right superior thyroid artery originating from the right external carotid artery in cadavers of a medical college in Nepal.

## METHODS

A descriptive cross-sectional study design was conducted to assess the variations on arterial supply of the thyroid gland in human cadavers in the dissection hall of Department of Anatomy, Universal College of Medical Sciences - Teaching Hospital, Bhairahawa, over the period of October 2020 to January 2021. Ethical approval was taken from Institutional Review Board of Universal college of Medical Sciences and Teaching Hospital, Bhairahawa, Rupandehi, Nepal (IRC UCMS, Ref: UCMS/IRC/078/20).

The sample size (n) was calculated as,

n = Z^2^ × p × q / e^2^

  = (1.645)^2^ × 0.9 × (1 - 0.9) / (0.1)^2^

  = 25

Where,

n = required sample size
Z = 1.67 at 90% Confidence Interval (CI)
p = 90%, prevalence from previous study^8^
q = 1-p
e = margin of error, 10%
Our calculated sample size was 25.

However,total thirty formalin embalmed cadavers of the department of anatomy were dissected exposing thyroid gland. Convenient sampling method was used. The cadavers who had any pathological lesions in neck region, cause of death were trauma to neck/chest was excluded for study. Variations in external carotid artery originated superior thyroid arteries was observed, recorded and photographed. All the collected data were entered into Microsoft Excel 2016. Point estimate at 90% Confidence Interval was calculated along with frequency and percentage.

## RESULTS

**Figure 1:**
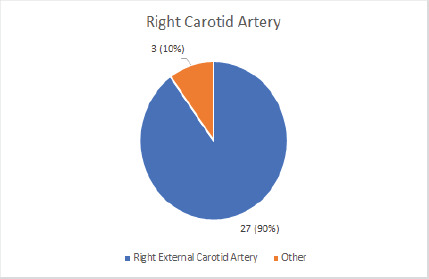
Origin of right superior thyroid arteries.

Out of 30 right superior thyroid arteries of 30 cadavers, 27 (90%) at 90% Confidence Interval (80.22-96.44) originates from right external carotid arteries (Figure 1).

Additionally, when we observed the sex distribution in origin of right superior thyroid artery it was 20 (90.90%) from right external carotid artery from 22 male cadavers and 7 (87.50%) from right external carotid artery from eight female cadavers (Table 1).

**Table 1:** Sex distribution for origin of right superior thyroid arteries from right external carotid arteries.

Sex	Right External carotid artery n (%)
Male (n=22)	20 (90.90)
Female (n=8)	7 (87.50)

Out of thirty right superior thyroid artery of 30 cadavers, 2 (3.33%) right superior thyroid artery have their origin other than external carotid artery. One (1.67%) right superior thyroid artery originate from carotid bifurcation (Table 2).

**Table 2:** Other origin of right superior thyroid arteries among 30 right superior thyroid arteries of 30 cadavers.

Origin of right STA	Right side n (%)
Carotid Bifurcation (CB)	2 (3.33)
Common carotid artery (CCA)	1 (1.67)

## DISCUSSION

Ranjit Sreedharan did a study in Kasturba medical college, Manipal and stated that 88.33% of STA originated from the ECA, 8.33% from the carotid bifurcation and 3.33% from common carotid artery which was quite similar to our study.^8^

In another study conducted by Dhindsa et al. and Shivaleela et al., STA arise from ECA in 66.67% in right side and 76.21% in left side, from common carotid bifurcation in 31.81% in right side, 21.43% in left side and from common carotid artery in 1.51% in right side, 2.38% in left side respectively.^9,10^ This was related from our study where STA arise from ECA in 90.0% from right side, carotid bifurcation 6.66% from right side, common carotid artery 3.3% from right side.

An digital subtraction angiographic study was done by pankaj gupta et al in 14 patients where 71.5% STA was originated from ECA in right side.^7^

In contrast to this MA Dessie did a study in 42 cadavers in ethopia that reported superior thyroid artery arises from the external carotid artery in 44.2%, carotid bifurcation in 27.9% and common carotid artery in 26.7%.^11^ Mean while Kevin Ongeti did a study in 46 cadaver in kenya in which he reported that superior thyroid artery arises 80% from external carotid artery, 13% from common carotid artery and 6.5 % from linguo-facial trunk of the cadavers.^12^

Kaan esen did a computer tomography study in 640 Japanese population and reported the right and left STA arose from the ECA in 64.5% and 39.7% patients, from the bifurcation of the common carotid artery in 20.5% and 23.1% patients, and from the common carotid artery in 14.1% and 35.3% patients, respectively.^13^ Similarly in another computer tomography study by Mario Herrera-Nunez, 50.7% of the STA was originated from ECA, 20.40% was originated from carotid body and 17.1% originated from common carotid artery respectively.^14^

Bergam reported that STA arise from the common carotid in 18% of cases, the point of division of the common carotid in 36%, or from the external carotid in 36% of cases.^15^

In our study, right STA originated more consistently from right ECA in male cadavers as compared to the female cadavers (90.9% vs 87.5%) which was similar to the finding a study by Toni, et al.^16^

Since the current study was carried out in 30 cadavers of a tertiary centre, the result of this study may not be generalized. However, study of similar type can be done in a large sample so that the actual findings can be generalized.

## CONCLUSIONS

The present study provided data of right ECA originated right STA which was high in our study as in comparison to previous other studies. These Variations plays an immense importance prior to planning of head and neck surgery.Thus, we recommend surgeons to have an extensive knowledge of variations prior to surgical innervations.
